# Design and applications of liposome-in-gel as carriers for cancer therapy

**DOI:** 10.1080/10717544.2022.2139021

**Published:** 2022-10-30

**Authors:** Yixuan Mou, Pu Zhang, Wing-Fu Lai, Dahong Zhang

**Affiliations:** a Urology & Nephrology Center, Department of Urology, Zhejiang Provincial People’s Hospital, Affiliated People’s Hospital, Hangzhou Medical College, Zhejiang, China; b Department of Applied Biology and Chemical Technology, Hong Kong Polytechnic University, Hong Kong Special Administrative Region, China

**Keywords:** Liposome-in-gel, cancer, nanomaterials, drug delivery

## Abstract

Cancer has long been a hot research topic, and recent years have witnessed the incidence of cancer trending toward younger individuals with great socioeconomic burden. Even with surgery, therapeutic agents serve as the mainstay to combat cancer in the clinic. Intensive research on nanomaterials can overcome the shortcomings of conventional drug delivery approaches, such as the lack of selectivity for targeted regions, poor stability against degradation, and uncontrolled drug release behavior. Over the years, different types of drug carriers have been developed for cancer therapy. One of these is liposome-in-gel (LP–Gel), which has combined the merits of both liposomes and hydrogels, and has emerged as a versatile carrier for cancer therapy. LP–Gel hybrids have addressed the lack of stability of conventional liposomes against pH and ionic strength while displaying higher efficiency of delivery hydrophilic drugs as compared to conventional gels. They can be classified into three types according to their assembled structure, are characterized by their nontoxicity, biodegradability, and flexibility for clinical use, and can be mainly categorized based on their controlled release, transmucosal delivery, and transdermal delivery properties for anticancer therapy. This review covers the recent progress on the applications of LP–Gel hybrids for anticancer therapy.

## 1. Introduction

GLOBOCAN (the World Health Organization’s International Agency for Research on Cancer Global Cancer Observatory) estimated that 19.3 million new cancer cases and 10.0 million cancer deaths occurred worldwide in 2020 (Sung et al., 2021). Cancer brings a great socioeconomic burden. Currently, surgery, radiotherapy, and chemotherapy remain the mainstays for cancer treatment, although immunotherapy and gene therapy have emerged as new methods to treat cancer. Because therapeutic agents involved in cancer treatment do not show sufficient accumulation in cancerous tissues and leak into normal tissues, these agents less effectively eradicate cancer and expose normal tissues to systemic toxicity (Pérez-Herrero & Fernández-Medarde, 2015). It is therefore urgent to develop novel drug delivery systems that accommodate therapeutic agents to tune the pharmacokinetics and biodistribution of the current drug options.

Liposomes are synthetic phospholipid nanovesicles with a bilayer membrane shape (Mao et al., 2016), whereas hydrogels are a class of extremely hydrophilic, three-dimensional network-structured gels. Both are widely used for delivering therapeutic agents to malignant tumors, and many aspects of their functions can be masked, including targeted delivery (Noble et al., 2014), sustained release, and resistance against degradation (Alinaghi et al., 2013). Their value is downplayed when they are used separately, and only the combination of liposomes and hydrogels is a win–win situation for all concerned issues (Table 1). Combining liposomes and hydrogels together generates a special type of drug delivery system, termed LP–Gel, which has shown better compatibility with more drugs and supports more functions for different uses. This review summarizes and discusses the recent progress in the application of LP–Gel for anticancer therapy.

**Table 1.** Advantages and disadvantages of liposomes, hydrogels, and LP–Gel.

Type	Disadvantages	Advantages	References
Liposome	Lack of stability against pH and ionic strength, uncontrolled release, and poor localized retention	Compatibility with hydrophobic and hydrophilic drugs, mucopenetration, and stimuli-responsiveness	(Alinaghi et al., 2013; Kazakov, 2016; GuhaSarkar et al., 2017)
Hydrogel	Incompatibility with lipophilic drugs, weak mucopenetration, and delicate structure	High stability, mucoadhesion, and prolonged drug release	(Furst et al., 2016; GuhaSarkar et al., 2017; Wu et al., 2018)
LP–Gel	/	Enhanced stability of liposomes supported by gel shells, better compatibility with hydrophobic and hydrophilic drugs, prolonged drug release kinetics, mucoadhesion to mucopenetration transition, and stronger mechanical support	(Furst et al., 2016; Kazakov, 2016; GuhaSarkar et al., 2017; Wu et al., 2018; Rahni & Kazakov, 2017)

## 2. Preparation of LP–Gel

Liposomes can be prepared by various methods, such as the thin film rehydration method, detergent removal method, injection method, extrusion method, heating method, supercritical fluid method, supercritical antisolvent (SAS) method, supercritical reverse-phase evaporation (SRPE) method, microfluidization, and ultrasonication. Regardless of their different preparation approaches, these processes follow a common pattern of three steps, which includes dissolution and dispersion in an organic solvent, addition of the lipid into an aqueous solution, and purification (Ajeeshkumar et al., 2021). Hydrogel preparation can be divided into physical crosslinking and chemical crosslinking. In more detail, the physical interactions are ionic interactions, hydrogen bonds, crystallization, hydrophobic interactions, and protein interactions. Chemical crosslinking is more common during hydrogel formation, as it supports better mechanical properties and stronger stability than physical crosslinking. Chemical crosslinking is dominated by conjugation reactions, free radical polymerization, and enzymatic reactions (Su et al., 2021).

**Figure 1.**
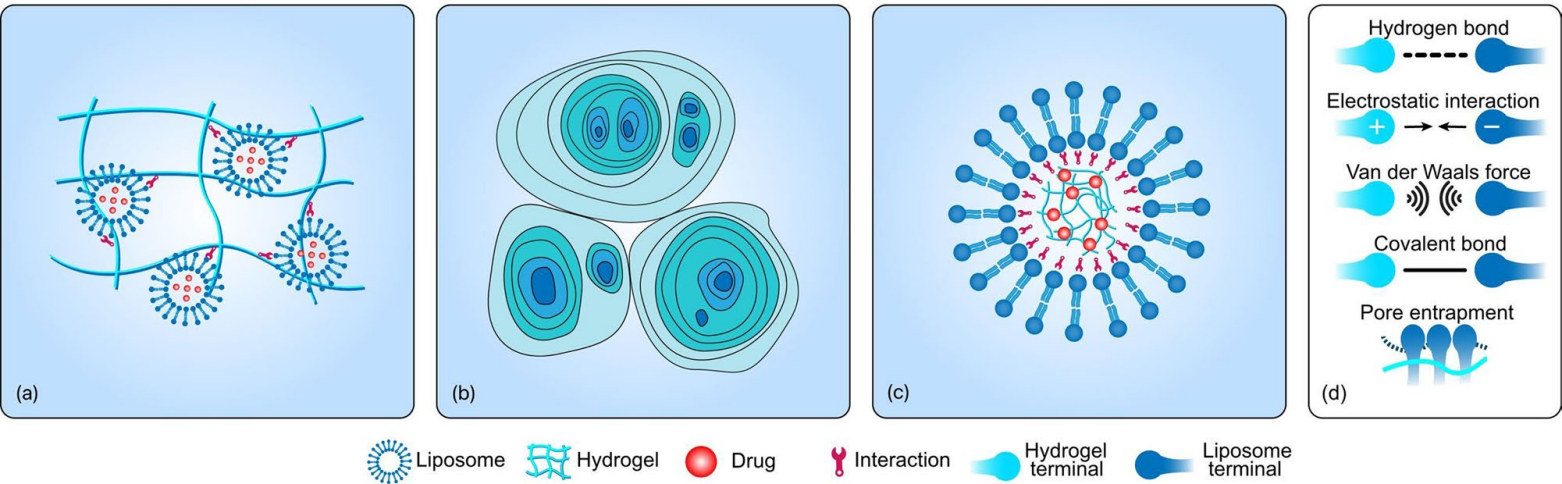
Types of LP–Gel hybrids and the interactions between the liposomes and hydrogels. (a) The incorporation of liposomes into hydrogel substrates via hydrogen bonds, van der Waals forces, electrostatic interactions, covalent binding, or pore entrapment. (b) Liposomes gelation to generate hydrogels via the electrostatic repulsive forces between bilayers. (c) Embedding of hydrogels into the core of liposomes via Coulombic attraction, covalent binding, or spontaneous phospholipidation of the gel. (d) Schematic diagram of each force.

To the best of our knowledge, the fabrication of LP–Gel hybrids can be classified into three categories (Figure 1): The incorporation of liposomes into hydrogel substrates, liposome gelation to generate hydrogels, and embedding hydrogels into the cores of liposomes. Most LP–Gel hybrids are created via the first method, which involves the sequential formation of liposomes and hydrogels. Liposomes and hydrogels can exist in two independent compartments, although interactions between them are always present. Liposomes can also show greater participation in the formation of hydrogels as one of their components. Jensen et al. introduced the assembly of LP–Gel hybrids via thiol-disulfide exchange between end group-modified poly(vinyl alcohol) and thiocholesterol-containing liposomes (Jensen et al., 2013). The process of liposome gelation can generate hydrogels, finally merging the hydrogels and liposomes into one component. Cheng et al. capitalized on the initial findings that at low bile salt/lecithin molar ratios in water, two classes of biological amphiphiles can self-assemble into hydrogels with microstructures made up of crowded, swollen multilamellar liposomes rather than the typical fibrous networks found in conventional hydrogels (Cheng et al., 2015). For these micro/nanosized hydrogels, the lipid envelope creates another type of LP–Gel hybrid, referred to as lipid-coated hydrogel nanoparticles. Both physical and chemical interactions are driving forces behind the absorption behavior of lipids onto the surface of micro/nanosized hydrogels (Raemdonck et al., 2014). However, the lipid coating steps of micro/nanosized hydrogels demand multiple rounds of separation. To alleviate this issue, unilamellar liposomes are first formed as a reactor for selective hydrogel formation in their aqueous lumen.

Therapeutic agents should be incorporated into LP–Gel hybrids to achieve the anticancer effect. Due to the physicochemical properties of LP–Gel hybrids, they are highly compatible with various therapeutic agents, among which chemotherapeutic agents are most widely used. The following sections will detail a combination of chemotherapeutic agents and LP–Gel hybrids. LP–Gel hybrids also serve as vehicles to carry biological drugs, such as small interfering RNA (siRNA) (Krebs & Alsberg, 2011; Furst et al., 2016; Ding et al., 2020), antibodies (Wang et al., 2021), cancer vaccines (Zhang et al., 2020), and enzymes (Bobone et al., 2015). siRNA-loaded LP–Gel hybrids showed high uptake efficiency in vaginal mucosa and could directly silence the expression of herpes viral proteins (Palliser et al., 2006; Furst et al., 2016). Chen et al. (2017) prepared liposome-templated hydrogel nanoparticles (LHNPs) for the codelivery of Cas9 protein and nucleic acids to fulfill a gene silencing function of the clustered regularly interspaced short palindromic repeat (CRISPR)/CRISPR-associated protein 9 (Cas9) technology. Trastuzumab (Tmab), as an antibody targeting human epidermal growth factor receptor 2 (HER2), was combined with LP–Gel hybrids to tackle breast cancer (Qin et al., 2018). In addition, neoantigen peptides, together with black phosphorus quantum dots, and immune adjuvants were incorporated into two compartments of LP–Gel to achieve photothermal immunotherapy (Zhang et al., 2020). Enzymes could also use LP–Gel hybrids to shield themselves from degradation and immune response (Bobone et al., 2015).

## 3. Design principles of LP–Gel

Colloidal drug delivery systems (CDDSs) have been widely investigated and used in drug delivery. Liposomes, as a CDDS, has attracted the most attention, mainly due to their amphiphilic nature, but also their advantages, including reduced toxicity, the capacity to load various drugs, and sustained drug release (Ali Khan et al., 2013). The barrier originating from the lipid bilayer membrane can create a rate-limiting effect on drug release. For example, the release of hydrophilic drugs follows the slow Higuchi release kinetic model initially and the zero-order kinetic model thereafter (Glavas-Dodov et al., 2002; Mourtas et al., 2007). However, low drug loading efficiency due to the tendencies of unwanted drug leakage, low stability in vivo, and low drug entrapment efficiency (EE%) limit the use of liposomes (Ali Khan et al., 2013; Petralito et al., 2014). Generally, an environment with pH values ranging from 2 to 6 and low ionic strength is favorable for maintaining the stability of liposomes with a high retention rate (RR) (Wang et al., 2020). However, the pH and ionic strength of body fluids and solid tissues in vivo are subject to change, especially under pathological conditions. For example, the pH of normal solid tissue is approximately 7.4, but becomes more acidic in tumor tissues (Lee & Thompson, 2017). For the intraluminal delivery of liposomes, the fluctuations in pH, ionic strength, and other physicochemical properties become much wider. Taking the bladder as an example, the pH of urine can change from 4.5 to 8.0 while the ionic strength remains high in general, which may lead to surface instability and liposome disruption. Panwar et al. demonstrated that the addition of a cholesterol component into the phospholipid bilayer did not change the site-specific relationship between the drug and the membrane. Instead, the addition of the cholesterol component enabled tight binding to phospholipid molecules, thus improving the stability and rigidity of the liposome (Panwar et al., 2010). However, Mohammadi et al. (2021) found that the addition of cholesterol into liposomes might result in the replacement of empty spaces with hydrophilic drugs. With further in-depth studies on hydrogels, their advantages regarding compatibility with liposomes have been discovered (Tabandeh & Mortazavi, 2013).

For liposomes in the hydrogel type, the hydrogel provides a protective layer for encapsulated liposomes to form the LP–Gel system, which can prevent the degradation of liposomal vesicles and drug leakage; thus, their stability is improved, and controlled release can be achieved (Lee et al., 2008; Moustafa et al., 2018). Embedding hydrogels into the core of liposomes can also bring better mechanical stability by providing a solid-like supporting cushion for the lipid bilayer (Kazakov, 2016). When LP–Gel was exposed to saline solutions at pH 7.4 or at pH 2.0, its porous structure remained stable, as indicated by scanning electron microscopy (GuhaSarkar et al., 2017). Overall, LP–Gel shows superior stability against external stimuli compared with liposomes and hydrogels. For liposomes in the hydrogel type, due to the steric hindrance of the hydrogels, LP–Gel can act as a sponge to slow the release of the encapsulated liposomes together with loaded drugs (Alinaghi et al., 2013). When drugs are delivered percutaneously, it is difficult for liposomes to adhere to the targeted region, while the gel formulation can wet the skin surface rapidly to boost transdermal penetration (Xu et al., 2022). When LP–Gel is used for intravesical instillation, hydrogels act as mucoadhesive substrates via covalent or noncovalent bonding, and they prevent the washout of drug-loaded liposomes in urine (De Geest et al., 2006). By the same principle, mucoadhesive LP–Gel can also prolong the retention of drug-loaded liposomes in the vagina (Furst et al., 2016).

For the lipid-coated hydrogel type, the hydrogel core can reduce the deformability of lipid bilayers and the resultant membrane fluidity, thereby promoting cellular uptake of drugs to enhance the cytotoxicity of chemotherapeutic drugs to cancer cells (Qin et al., 2018). Besides, the hydrogel core can respond to external stimuli and either swells or shrinks in a reversible and controllable process, finally resulting in mechanical squeezing of the loaded drug (De Geest et al., 2006; Krebs & Alsberg, 2011). The drug release properties of LP–Gel are dominated by multiple factors (Kazakov, 2016). Fathalla et al. incorporated liposomes into different hydrogels, including Pluronic® F-127 (PL-127), hydroxyethyl cellulose (HEC), and hydroxypropyl methylcellulose (HPMC), and compared their drug release kinetics. The drug release rates after 24 h ranked as follows: HEC gel > PL-127 gel > HPMC gel (Fathalla et al., 2020). The viscosities of the PL-127, HPMC, and HEC gels were 720 ± 9.1, 100 ± 5.1, and 40 ± 2.1 Pa s, and the leading role in determining the drug release rate was doubtful. Fathalla et al. indicated other confounding factors, including gel dissolution rates and specific interactions between the drug and polymers in the gel. In addition, the concentration of polymer used for building hydrogels modulates drug release by altering the viscosity and diffusion coefficient (Ricci et al., 2005). The liposomal component is deemed another determiner that regulates the drug release kinetics. Liposomes generated from different lipids, including Phospholipon 90G and Lipoid S100, were assembled into poloxamer gels, and their drug release rates after 8 h were calculated to be 71.6 ± 3.28% and 54.4 ± 4.26%, respectively (Tanrıverdi et al., 2016). Mourtas et al. prepared two kinds of drug-loaded liposomes using phosphatidylcholin (PC) or distearoyl-glycero-PC with the addition of cholesterol to obtain DSPC/Chol, which was then transferred to a combination with carbopol 974 hydrogels or HEC hydrogels (Mourtas et al., 2007).

Drug release-related features should be considered when loading drugs with different physicochemical properties. The release of lipophilic drugs from LP–Gel is dependent on the total amount of drug in the gel and is independent of the rigidity of the above two types of liposomes; however, the rigidity of the liposomal membrane is the most important factor that determines the release rate of hydrophilic drugs. Further studies on the comparison between the aforementioned two gels revealed that the carbopol hydrogel permitted faster drug release kinetics than the HEC hydrogel, which might be related to their different rheological properties. The rheological properties of LP–Gel, in turn, are related to the doped concentration, composition (Hurler et al., 2013), and surface charge (Boulmedarat et al., 2003) of the liposomes. Boulmedarat et al. indicated that loading positively charged liposomes with lipid up to a concentration of 10 mM significantly promoted the viscosity of carbomer hydrogels (Kazakov, 2016). Negatively charged liposomes are able to interact with hydrogels to slightly reduce the gelation rate and gel strength (Ruel-Gariépy et al., 2002). Incorporating liposomes with high lipid concentrations within a certain range can increase the gel viscosity. Compared to liposomal hydrogels composed of glycerol or isopropyl alcohol, the viscosity of liposomal hydrogels containing propylene glycol decreases because propylene glycol may dissolve the phospholipids to diminish the consistency of the hydrogels (Dejeu et al., 2022).

Hydrogels are commonly used for topical administration due to their adhesive properties, but most of them are compatible with only hydrophilic drugs rather than lipophilic drugs. Lipophilic drugs, such as paclitaxel (PTX) (GuhaSarkar et al., 2017), have low solubility in water but high affinity for the hydrophobic lipid bilayers of liposomes, allowing them to be encapsulated within the lipid bilayers. Due to their soft physical properties and relatively large size, hydrogels exhibit a weak ability to penetrate into tumors. Hydrogels enable the prolonged retention and longer duration of action of drugs in targeted regions, whereas mucus-penetrating liposomes can be packed into hydrogels to support the mucoadhesion-to-mucopenetration transition, which ensures the penetration of drugs deep into the targeted regions. The combination also strengthens the mechanical support of the hydrogel (Yazar et al., 2004). Wu et al. (2018) developed a gemcitabine-loaded LP–Gel for the sustained release of gemcitabine for the long-term treatment of osteosarcoma in situ. Importantly, the addition of liposomes into the hydrogel greatly strengthened the hydrogel, and the complete LP–Gel could be used as an excellent packaging material for tissue regeneration. With increasing liposome content, the compressive modulus of the complete LP–Gel increases and becomes threefold stronger than that of pure gelatin methacrylate. Another concerning issue is the steric hindrance that the passively diffusing liposomes in the hydrogel are exposed to. However, these encapsulated liposomes are capable of migrating faster than other liposome-based complexes, and the particle size of the liposome has a great impact on their diffusion efficiency (Furst et al., 2016). For lipid-coated hydrogels, the shielding of the hydrogel by a lipid layer can provide better biocompatibility and bioavailability in vivo. Moreover, this shielding also preserves the responsiveness of liposomes to external stimuli with a reduced possibility of drug leakage (Kazakov, 2016; Rahni & Kazakov, 2017). The pros and cons of liposomes, hydrogels, and LP–Gel mentioned earlier are summarized in Table 1.

The mechanism of binding between liposomes and hydrogels is still not clear. It has been speculated that because the pore sizes of the hydrogels are smaller than those of liposomes, the liposomes are easily trapped (Varghese et al., 2014). Contrary to this belief, Thompson et al. proposed that interchain bonds may involve entanglement (topological constraints) together with weak physical interactions, such as van der Waals forces and hydrogen bonding. Physical interactions are always present and may modulate the pore size through dynamic connection changes. Analysis of fourier transform infrared (FTIR) spectra and differential scanning calorimetry (DSC) thermograms indicated that hydrogen bonds are formed between liposomes and hydrogels (Alinaghi et al., 2014; Liu et al., 2020). Nitrogen atoms in hydrogels can participate in hydrogen bonds with some elements in liposomes, such as phosphorus. These hydrogen bonds can stabilize hydrogels by twisting the hydrogel network to produce a double-crosslinked structure (Chen et al., 2012). There are two mechanisms responsible for the release of liposomes from hydrogels. First, the crosslinks in the hydrogel must be dynamic so that the pore size can change to induce the release of liposomes. Moreover, liposomes must be sufficiently flexible and deformable to ensure that they can squeeze through the pores (Thompson et al., 2020). For lipid-coated hydrogels, the introduction of hydrophobic anchors allows the self-assembly of the phospholipid bilayers to adsorb on the gel surface, or Coulombic attraction between the charged microgels and oppositely charged lipids can promote the assembly of the lipids onto the hydrogel core (Raemdonck et al., 2014; Kazakov, 2016). In addition, other novel mechanisms exist to trigger their assembly. Liposomes can also be partially adsorbed onto the hydrogel surface and partially internalized by diffusion due to their deformability (Youssef et al., 2013).

## 4. Applications for anticancer therapy

**Figure 2.**
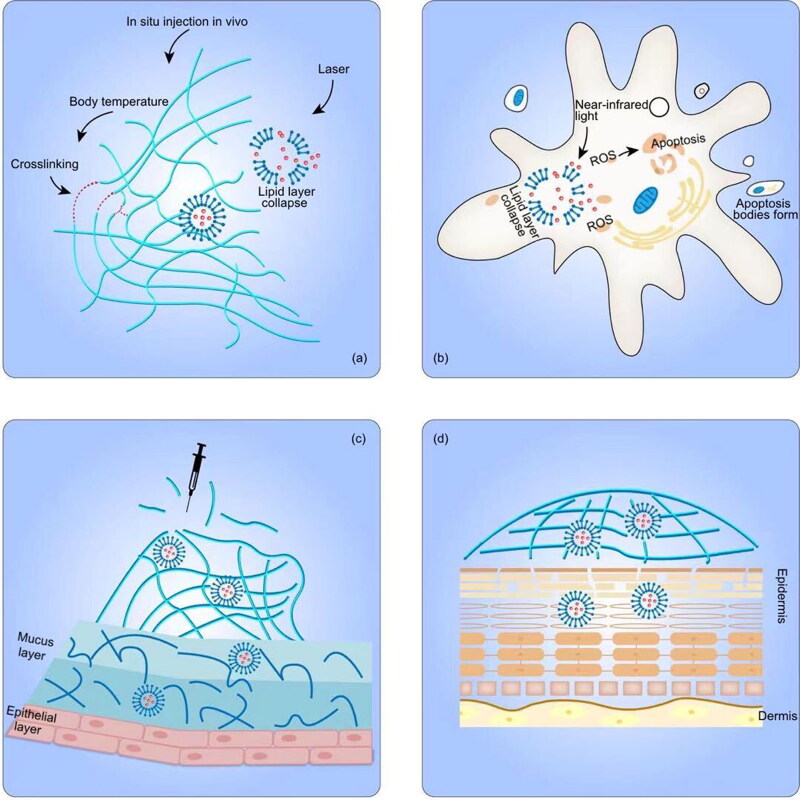
Different properties of LP–Gel in anticancer therapy. (a) Thermosensitive LP–Gel, (b) photosensitive LP–Gel, (c) transmucosal LP–Gel, and (d) transdermal LP–Gel.

As far as we know, there still lacks clinical use and clinical trials relevant to the anticancer application of LP–Gel hybrids **(**Table 2 and Figure 2**)**. More details on clinical trials of LP–Gel hybrids for other diseases, can be found in Supplementary Table S1.

**Table 2.** Applications of LP–Gel for anticancer therapy.

Drug	Tested models (cell line(s)/animals)	Components of LP–Gel	Mechanism	Performance	Ref.
PTX	NBT-II cells, T24 cells/Wistar rat	SPC-liposome, gellan hydrogel	Urine-triggered cross-linking of the gel to enhance its adhesion to the mucus layer followed by PTX-loaded liposomes diffusion into the attached region	Threefold increase in the mucoadhesiveness of the liposomes in LP–Gel compared with that of free liposomes and prolonged retention periods up to 7 days; in vivo anticancer therapy data is lacking	(GuhaSarkar et al., 2017)
Cur	MCF-7 cells/nude mice	PC/Chol-liposome, chitosan hydrogel	Gelation at 37 °C in vivo to achieve localized and sustained release	Slow degradation in vivo; more pronounced inhibition of the growth of MCF-7 cell-derived breast cancer without recurrence and lung metastasis over 24 days compared to free liposomes	(Li et al., 2020)
Rap	5637 cells, HT-1376 cells, MBT2 cells/ C3H mice	SPC/Chol-liposome, P407 hydrogel	Same as above	Tumor inhibition effect (up to 40%) induced by low-dose Rap exposure (1 μg) for 48 h	(Yoon et al., 2019)
PTX	B16F10 cells/ C57BL/6 mice	SPC-liposome, gellan hydrogel	Same as above	Higher tumor inhibition rate (84.3 ± 4.2%) than that after radiation alone (75.5 ± 4.1%)	(GuhaSarkar et al., 2016)
Tmab	Drug-resistant HER2 cells, SK-BR-3 cells/nude mice	PC/PE/Chol-liposome, ALD- XA gydrogel	Oxidation of lipids by the ROS generated from Ce6 under NIR light to interrupt the integrity of liposomes	Upregulated the release rate of Tmab up to 10.36 ± 0.49% under NIR stimulation for 180 s; improved tumor suppression and ablation efficiency	(Wang et al., 2021)

Abbreviations: PTX: paclitaxel, can be used as radiosensitizer; NBT-II: a rat bladder cancer cell line; T24: a human bladder cancer cell line; SPC: soy phosphatidylcholine; Cur: curcumin; MCF-7 cells: a human breast cancer cell line; PC: phosphatidylcholine; PE: phosphatidylethanolamine; Chol: cholesterol; Rap: rapamycin; 5637 and HT-1376: human bladder cancer cell lines; MBT2: a mouse bladder cancer cell line; B16F10: a murine melanoma cell line; Tmab: trastuzumab; SK-BR-3: a human breast cancer cell line; ROS: reactive oxygen species; Ce6: photodynamic component; NIR: near-infrared.

### 4.1. Stimuli-responsive LP–Gel hybrids

The incorporation of stimuli-responsive liposomes into hydrogels constitutes one type of stimulus-responsive LP–Gel. Taking advantage of the thermosensitivity and feasibility of injection in situ, LP–Gel hybrids can be made to exist in a sol state at room temperature and be converted into a solid gel state in situ upon contact with the physical environment in vivo. Amphiphilic triblock PLGA–PEG–PLGA copolymers have been used to prepare thermosensitive hydrogel formulations, and the gel activation that occurs when the temperature reaches 37 °C results in micelle aggregation by increasing hydrophobic interactions between the PLGA moieties (Li et al., 2014). Over time, at body temperature, the in situ gel slowly degrades, and the drug-loaded liposomes are then slowly released (Cao et al., 2019). Similarly, Mao et al. prepared thiolated chitosan-coated liposomes, which were fluidic at room temperature but gelled quickly at 37 °C, to deliver curcumin to breast cancer cells (Mao et al., 2016; Li et al., 2020). Thermosensitive liposomes embedded in thermosensitive hydrogels provide a more flexible way to control drug release and enhance the localized anticancer effects. The NIR-II photothermal agent DPP-BTz and gemcitabine were encapsulated in thermosensitive liposomes and then combined with the hydrogel precursor solution (Kong et al., 2021). The hydrogel precursor solution carrying drug-loaded liposomes was injected into pancreatic tumors and then converted into a crosslinked gel structure. Under laser irradiation, DPP-BTz can generate heat to break the liposome shell, allowing drug release. Sugiyama et al. used this approach to deliver a liposomal temperature-sensitive gel (PLTG) containing paclitaxel to ovarian cancer-originating seeding metastases via intraperitoneal injection, guaranteeing the accumulation of drug in the peritoneal cavity. Upon contact with the in vivo environment, the increased temperature elicited the formation of a solid LP–Gel, which was retained in the peritoneal cavity (Sugiyama et al., 2022). Compared with the paclitaxel-loaded temperature-sensitive gel PTG, the sustained release capability of PLTG not only prolonged the duration of drug action but also reduced the amount of free paclitaxel exposed to the abdominal cavity, thus avoiding side effects (Chambers et al., 2012). Aside from chemotherapeutic agents, thermosensitive LP–Gel can also be used for photothermal immunotherapy. As proposed by Zhang and coworkers (Chen et al., 2021), colon cancer cell-derived neoantigen peptides, as cancer vaccines, together with black phosphorus quantum dots were co-encapsulated into liposomes, which were then loaded into F12 gel containing immune adjuvants. Under 808 nm near-infrared laser irradiation, heat generated from black phosphorus quantum dots could accelerate the gel ablation, allowing the subsequent release of immune adjuvants and liposomes to stimulate an immune response against cancer (Chen et al., 2021).

A photosensitive LP–Gel composed of photosensitive liposomes and hydrogels shares the same mechanism of responsive drug release with photosensitive liposomes. Under light excitation at a specific wavelength, the photosensitizers embedded in photosensitive LP–Gel generate reactive oxygen species (ROS), which react oxidatively with unsaturated phospholipid materials to disrupt liposomes. The generated ROS localized in the LP–Gel-attached tumor regions can also induce tumor cell apoptosis or necrosis for promising potential in photodynamic therapy. Chen et al. encapsulated a tumor-targeting photosensitizer, IR780, into liposomes, and then incorporated the liposomes into hydrogels. This hybrid system was applied for the systematic delivery of photosensitizers to subcutaneous tumors and deep metastatic sites via topical administration (Nasr et al., 2019). Analogously, an experimental gel containing liposomal hydroxyl-aluminum phthalocyanine was used for topical photodynamic therapy to treat mammary carcinoma (Sutoris et al., 2012). Wang et al. (2021) introduced a combination of photodynamic therapy and monoclonal antibody therapy. Liposomes containing the photosensitizers chlorin (Ce6) and trastuzumab were prepared into hydrogels, and the final product exhibited an excellent shear response and near-infrared light-triggered drug release.

Lipid-coated hydrogels can also be stimuli-responsive. To prevent drug leakage and microgel swelling, Kiser et al. coated a drug-loaded microgel with a lipid bilayer to form lipobeads to promote permeability into the tumor vasculature (Kiser et al., 1998, 2000). During drug release, an electroporating pulse was initially used to perforate the lipid bilayer, exposing the enveloped microgel to water, causing it to swell. Electroporation and gel swelling resulted in disruption of the lipid bilayer (De Geest et al., 2006). The next step was drug release from the microgel. The properties of polymer networks conferred their responsiveness to environmental stimuli, such as temperature and pH. When the environment changes, the polymer network can also shrink like a sponge and squeeze the drugs into the space between the gel and lipid membrane, leading to drug diffusion across the lipid membrane (Kazakov, 2016). In conclusion, lipobeads maintain the advantages of liposomal drug carriers while additionally providing stronger mechanical support with better stimuli responsiveness. Furthermore, the increased stiffness conferred by complexation of the microgel greatly enhanced the cellular uptake of the lipobeads (Qin et al., 2018).

### 4.2. Biobarrier penetrating LP–Gel hybrids

Due to mucus clearance mechanisms and the intraluminal protective barrier, foreign materials hardly reach the inner region of tissues with mucus linings. LP–Gel is a hybrid system in which mucoadhesion and mucopenetration can coexist independently, fitting well with the demand of intraluminal drug delivery. For applications in the bladder, GuhaSarkar et al. introduced an LP–Gel consisting of paclitaxel-loaded fluidizing liposomes and gellan hydrogels. Gellan hydrogels can be triggered by ions present in the urine and crosslinked to form an urothelium-adherent gel, and the fluidizing liposomes can fluidize the urothelial barrier for enhanced penetration (Hu et al., 2009). Yoon et al. prepared folate-modified rapamycin (Rap)-loaded liposomes into poloxamer 407-based hydrogels. The poloxamer 407-based hydrogel formed a gel inside the bladder after exposure to body temperature and prolonged the retention time of the liposomes. Folate modification improved the receptor-mediated endocytosis and tumor targeting efficiency (Yoon et al., 2019). In addition, LP–Gel hybrids are an excellent choice for drug delivery in the vaginal and cervical regions. A polyethylene glycol-modified lipid complex containing siRNA was encapsulated in a solid matrix (formed by freeze-drying a hydrogel, called a sponge) to prolong its action in the vaginal mucus, and pegylated lipoplexes acted as mucus-penetrating vehicles to migrate deep into the vagina (Ali Khan et al., 2013). Li et al. prepared a novel expandable hydrogel foam aerosol delivery system with propylene glycol-embedded liposomes (PEHFLs) for vaginal drug delivery. The PEHFL foam gradually expands after spraying, and the size after expansion is influenced by the ambient temperature, which greatly enhances the uniformity of drug diffusion into the vagina and promotes mucoadhesion to the vagina (Li et al., 2012).

Liposomes are more rigid in nature than other ultraflexible forms of lipid vesicles, such as transferosomes, ethosomes, and transethosomes, so liposomes are less effective for transdermal drug delivery (Sudhakar et al., 2021). Another major disadvantage of liposomes lies in the liquid nature of their preparation. In this respect, LP–Gels can be used as high-viscosity vehicles to retain liposomes for transdermal delivery and can weaken the barrier function of the stratum corneum (SC) without notable changes to the dermis and subcutis (Guan et al., 2015). In accordance with this principle, the amount of accumulated IR780 in tumor regions after topical administration onto tumors was nearly equal to that after intravenous administration, and the amount applied via topical administration was approximately three-quarters that administered intravenously (Chen et al., 2021). Lipid-coated nanogels can also reside in the gel to form a more complex LP–Gel. Nasr et al. embedded lipid-coated nanogels carrying ferrous chlorophyllin (PC/CHI) into gels to treat squamous cell carcinoma via topical administration onto tumors. Compared to the free form of ferrous chlorophyllin, PC/CHI increased about 10-fold retention of ferrous chlorophyllin on the skin. However, the penetration depth of PC/CHI was limited to the epidermis due to the rigid structure of lipid-coated nanogels (Nasr et al., 2019).

## 5. Comparison with other nanomaterials

The most important concerns to be met for anticancer drug delivery systems are the tumor regions being exposed to high concentrations of drugs and optimal drug release behavior. In the following section, comparisons of these two features, localized enrichment (Table 3) and controlled release (Table 4), between LP–Gel and other nanomaterials will be discussed.

**Table 3.** Comparison of LP–Gel and other nanomaterials regarding localized enrichment therapy.

Nanomaterial	Drug	Size (nm)	Zeta potential (mV)	Driving force for enrichment	Performance	Disadvantages (compared with LP–Gel)	Ref.
MNPs	Citric acid	163.2 ± 2.4	16.3 ± 3.7	External magnetic field	Increased cellular uptake in targeted regions	Damage to normal cells; the magnetic field strength needs to be adjusted to fit each individuals; inconvenient to carry	(T S et al., 2020)
Liposomes	DXR	<400	–	EPR effect	Accumulation in tumors at concentrations 5–10 times higher than that in plasma	Influenced by cancer heterogeneity; entirely dependent on passive diffusion and the liposome particle size	(Gabizon et al., 2006; Duncan et al., 1996)
HA-coated liposomes	GEM	192 ± 2	43 ± 1	Targeting ligands	Nearly 9-fold increase in tumor accumulation compared with that of naked liposomes	Influenced by the surface ligand coating density; elimination of targeting capability after protein corona formation followed by the activation of the MPS	(Arpicco et al., 2013)

Abbreviations: MNP: magnetic nanoparticles; DXR: doxorubicin; HA: hyaluronic acid (32 disaccharidie units); GEM: gemcitabine; MPS: mononuclear phagocytic system.

**Table 4.** On-demand release mechanisms of other nanomaterials.

Thermosensitive MLs	CF	AMF	MNPs	The heat generated from MNPs above the Tm, together with mechanical stress, increases the membrane permeability.	(Spera et al., 2015)
Photosensitive liposomes	Calcein	UV light	MM	Unidirectional molecular rotation under UV light disturbs the lipid bilayer to increase the membrane permeability.	(Ribovski et al., 2020)
Photosensitive liposomes	Pt(IV)	650 nm light	Ce6	Oxidation of lipids by the ROS generated from Ce6 under NIR light interrupts liposomal integrity	(Yang et al., 2021)
Photothermal liposomes	DOX	NIR laser	ICG	Heat generated from ICG under NIR laser above the Tm increases the membrane permeability.	(Dai et al., 2019)

Abbreviations: MLs: magnetoliposomes; CF: 5-carboxyfluorescein; AMF: alternating magnetic field; MNPs: superparamagnetic iron oxide nanoparticles; *T*_m_: lipid melting point; MM: molecular motor; Pt(IV): tetravalent platinum prodrug; Ce6: photodynamic component; ROS: reactive oxygen species; ICG: indocyanine green; NIR: near-infrared; PCMs: phase change material.

Under the guidance of an external magnetic field, magnetic nanoparticles (MNPs) can actively target tumors and release the loaded drugs on demand. When magnetic nanoparticles are complexed with liposomes, the so-called magnetic liposomes (MLs) can reduce the toxicity of MNPs while inheriting the magnetic-responsive properties (Li et al., 2018; Madan et al., 2019; T S et al., 2020). Kono et al. coated MLs with atelocollagen to improve cellular uptake efficiency. After intravenous injection of atelocollagen-coated MLs, a disc-shaped magnet was placed close to the liver to accelerate ML enrichment inside the liver (Kono et al., 2017). Another study showed that magnetic anionic liposome (Mag-AL) binding was significantly increased under magnetic guidance in RAW264 cells (a murine macrophage-like cell line) compared to that of the control group. Moreover, the magnetic field-guided cell binding and uptake of liposomes increased linearly with increasing liposome concentration over a wide range (0–50 mg/mL) (Yatvin et al., 1978). However, long-term exposure to a magnetic field may damage normal cells (Jain et al., 2021), as normal cells carrying MLs may detach from where they are supposed to be. In addition, the magnetic field strength needs to be adjusted according to the thickness of each individual’s skin and amount of subcutaneous fat. It is also inconvenient to carry magnetic-field-generating devices for long-term therapy. Because of proximal tumor vascular leakage and impaired lymphatic drainage within the tumor microenvironment, liposomes can passively target tumor cells through an enhanced permeation and retention effect (EPR effect) (Gabizon et al., 2006). However, the capacity of passive targeting is entirely dependent on diffusion-regulated drug transport mechanisms and is dependent on the particle size of the liposomes (Dreher et al., 2006). Moreover, it has been demonstrated that the EPR effect is highly variable in human patients due to the heterogeneity of cancer cells (Matthew et al., 2021). Therefore, it is necessary to endow liposomes with active targeting capabilities.

Recently, Arpicco et al. proposed hyaluronic acid (HA) to act as a targeting ligand after its immobilization onto the surface of liposomes containing gemcitabine. HA specifically binds the cell surface receptor CD44, which is overexpressed in various tumors. HA is also chemically modifiable and biodegradable. HA-coated liposomes target pancreatic cancer cells expressing CD44, and the amount of HA (32 disaccharide units)-coated liposomes internalized was quantified and determined to be nearly ninefold more than that of naked liposomes at 24 h (Arpicco et al., 2013). Peptides, antibodies, and aptamers have great potential to be used for surface modification of liposomes with targeting moieties, but many factors, such as particle size, surface charge, ligand density, and ligand conformation, should be carefully considered (Yan et al., 2020). For instance, liposomes coated with cationic targeting ligands are preferred drug delivery systems due to their strong interaction with the tumor cell membrane. Optimizing the ligand density on the surface of the liposomes allows enhanced internalization by tumor cells, but liposomes may aggregate once the surface density exceeds the optimum value. In addition, the formation of a protein corona is inevitable once liposomes are exposed to the circulatory system (Onishchenko et al., 2021). The protein corona contributes to the thickness of the hydrodynamic radius of the liposomes and the normalization of the liposomal zeta potential at approximately –20 mV. Physicochemical changes to the particle surface eliminate the targeting effect produced by surface-anchored ligands. Unfortunately, the pharmacokinetics and biodistribution are altered as undesirable side effects after the protein corona containing opsonins forms. The mononuclear phagocytic system (MPS) is then activated to remove liposomes from circulation (Noble et al., 2014; Ross et al., 2018).

When the temperature reaches a value above the thermosensitive lipid melting point (*T*_m_), the permeability of the liposomal membrane increases and then the drugs are released without destroying the liposome structure. Heating to temperatures over the *T*_m_ allows on-demand drug release (Yatvin et al., 1978). MLs, when exposed to a magnetic field of appropriate frequency, can generate heat, which is mainly derived from self-hysteresis consumption or from Néel or Brownian relaxation processes (Spera et al., 2015). The magnetically induced heat can serve as a switch to trigger on-demand drug release, but its drawbacks, such as inconvenience for daily use, have limited its further clinical applications (Haša et al., 2018). The mechanism of on-demand drug release may also be related to mechanical stimulation by a low-amplitude magnetic field conferring mechanical stress on the liposomal membrane, causing nearby nanoparticles to oscillate and thus triggering drug release (Spera et al., 2015). The incorporation of photodynamic hydrophobic molecular motors into a special type of liposomes known as molecular motor (MM) liposome complexes can facilitate on-demand drug release via the mechanism of molecular rotation. Upon UV irradiation, the MM undergoes photoisomerization around the central double bond and a subsequent thermal relaxation step, activating unidirectional molecular rotation and enhancing the permeability of the liposomal membrane (Ribovski et al., 2020). Yang et al. prepared photoactivated liposomes containing the photodynamic component Ce6. The ROS generated from Ce6 after photoactivation can oxidize unsaturated phospholipids into hydrophilic peroxides and thus destabilize liposome integrity. Since oxidization is reversible, repetitive controlled drug release can be achieved (Yang et al., 2021). Regarding photothermal liposomes, indocyanine green (ICG) was combined with thermosensitive liposomes. The ICG can absorb near-infrared radiation, which is then translated into a thermal effect to melt liposomes (Dai et al., 2019).

## 6. Conclusions and prospects

In summary, this review covers the progress in LP–Gel research. LP–Gel hybrids are nontoxic, biodegradable, biocompatible, and can undergo chemical modification. In addition, they are hybrid systems composed of liposomes and hydrogels that can overcome the drawbacks of one single composition type. To the best of our knowledge, LP–Gel hybrids are mainly used for localized anticancer therapy, and can be further categorized based on their controlled release, transmucosal delivery, or transdermal delivery properties. Regarding their unique features for anticancer therapy, a comparison between LP–Gel hybrids and other commonly used nanomaterials was presented. Overall, LP–Gel is a promising drug delivery system that has great potential for further clinical use.

However, from our own perspective, several questions regarding the biosafety remain to be answered. During intravesical instillation, LP–Gel may be an obstacle to the urine flushing out of the bladder, ultimately leading to renal dysfunction. For solid tumors that are unreachable via intraluminal administration, in situ injection into tumors via needle-related administration may trigger the spread of tumor cells. The next question comes down to whether LP–Gel can advance its functions to be more effective and flexible for anticancer therapy. Regarding on-demand release, nearly all studies place a great focus on incorporating stimuli-responsive liposomes into hydrogels, which mainly serve as simple substrates. It is believed that stimulators can also be equipped into high-density hydrogels, and effectors, such as heat and ROS, can be rapidly transferred onto the liposomal surface. Even for more rapid response to external stimuli, stimulators can reside in both liposomes and hydrogels, and furthermore, different types of stimulators can also be packed into LP–Gel so that dual-stimuli response can be achieved. For those LP–Gels buried deep in tissues, most stimuli are blocked by the hindrance of tissues. Taken together, novel stimulators should be considered for the modification of LP–Gel. For instance, upconversion nanoparticles (UCNPs) are capable of converting multiple near-infrared (NIR) photons to ultraviolet or visible photons via an anti-Stokes mechanism. UCNPs have been applied to loaded in liposomes or hydrogels for on-demand release via tissue-penetrating laser stimulation (Huang et al., 2016; Yao et al., 2016; Liu et al., 2020; Mehata et al., 2020). The ultrasound (US)-responsive stimulator is another choice, and it can be designed as US-responsive hydrogels or liposomes (Yue et al., 2019; Meng et al., 2021). As for biobarrier penetrating LP–Gel hybrids, their application is limited to the localized therapy. For most types of cancers without natural cavities connecting with the outside, LP–Gel works in vain. However, the lipid-coated hydrogel may be allowed for systemic therapy via intravenous administration. Like other-drug delivery systems, its targeting capacity can be gained by functionalizing its surface with targeting ligands or modulating its passive targeting property via EPR effect. Therapeutic agents can be loaded into the hydrogel core or the interspace between the liposome membrane and the hydrogel core, and get released once the endocytosis of the lipid-coated hydrogel helps rupture the shielding liposome membrane.

## Supplementary Material

Supplemental Material

## References

[CIT0001] Ajeeshkumar KK, Aneesh PA, Raju N, et al. (2021). Advancements in liposome technology: preparation techniques and applications in food, functional foods, and bioactive delivery: a review. Compr Rev Food Sci Food Saf 20:1280–306. 33665991 10.1111/1541-4337.12725

[CIT0002] Ali Khan A, Mudassir J, Mohtar N, et al. (2013). Advanced drug delivery to the lymphatic system: lipidbased nanoformulations. Int J Nanomed 8:2733–44. 10.2147/IJN.S41521 PMC3732201 23926431

[CIT0003] Alinaghi A, Rouini MR, Johari Daha F, et al. (2013). Hydrogel-embeded vesicles, as a novel approach for prolonged release and delivery of liposome, in vitro and in vivo. J Liposome Res 23:235–43. 23697905 10.3109/08982104.2013.799179

[CIT0004] Alinaghi A, Rouini MR, Johari Daha F, et al. (2014). The influence of lipid composition and surface charge on biodistribution of intact liposomes releasing from hydrogel-embedded vesicles. Int J Pharm 459:30–9. 24239579 10.1016/j.ijpharm.2013.11.011

[CIT0005] Arpicco S, Lerda C, Dalla Pozza E, et al. (2013). Hyaluronic acid-coated liposomes for active targeting of gemcitabine. Eur J Pharm Biopharm 85:373–80. 23791684 10.1016/j.ejpb.2013.06.003

[CIT0006] Bobone S, Miele E, Cerroni B, et al. (2015). Liposome-templated hydrogel nanoparticles as vehicles for enzyme-based therapies. Langmuir 31:7572–80. 26102092 10.1021/acs.langmuir.5b01442

[CIT0007] Boulmedarat L, Grossiord JL, Fattal E, et al. (2003). Influence of methyl-beta-cyclodextrin and liposomes on rheological properties of Carbopol 974P NF gels. Int J Pharm 254:59–64. 12615410 10.1016/s0378-5173(02)00683-x

[CIT0008] Cao D, Zhang X, Akabar MD, et al. (2019). Liposomal doxorubicin loaded PLGA-PEG-PLGA based thermogel for sustained local drug delivery for the treatment of breast cancer. Artif Cells Nanomed Biotechnol 47:181–91. 30686051 10.1080/21691401.2018.1548470

[CIT0009] Chambers SK, Chow HH, Janicek MF, et al. (2012). Phase I trial of intraperitoneal pemetrexed, cisplatin, and paclitaxel in optimally debulked ovarian cancer. Clin Cancer Res 18:2668–78. 22421191 10.1158/1078-0432.CCR-12-0261 PMC3343173

[CIT0010] Chen D, Sun K, Mu H, et al. (2012). pH and temperature dual-sensitive liposome gel based on novel cleavable mPEG-Hz-CHEMS polymeric vaginal delivery system. Int J Nanomed 7:2621–30. 10.2147/IJN.S31757 PMC3368511 22679372

[CIT0011] Chen G, Ullah A, Xu G, et al. (2021). Topically applied liposome-in-hydrogels for systematically targeted tumor photothermal therapy. Drug Deliv 28:1923–31. 34550040 10.1080/10717544.2021.1974607 PMC8462874

[CIT0012] Chen Z, Liu F, Chen Y, et al. (2017). Targeted delivery of CRISPR/Cas9-mediated cancer gene therapy via liposome-templated hydrogel nanoparticles. Adv Funct Mater 27:1703036. 29755309 10.1002/adfm.201703036 PMC5939593

[CIT0013] Cheng CY, Wang TY, Tung SH. (2015). Biological hydrogels formed by swollen multilamellar liposomes. Langmuir 31:13312–20. 26574777 10.1021/acs.langmuir.5b03267

[CIT0014] Dai Y, Su J, Wu K, et al. (2019). Multifunctional thermosensitive liposomes based on natural phase-change material: near-infrared light-triggered drug release and multimodal imaging-guided cancer combination therapy. ACS Appl Mater Interfaces 11:10540–53. 30807086 10.1021/acsami.8b22748

[CIT0015] De Geest BG, Stubbe BG, Jonas AM, et al. (2006). Self-exploding lipid-coated microgels. Biomacromolecules 7:373–9. 16398538 10.1021/bm0507296

[CIT0016] Dejeu IL, Vicaș LG, Vlaia LL, et al. (2022). Study for evaluation of hydrogels after the incorporation of liposomes embedded with caffeic acid. Pharmaceuticals (Basel) 15:175. 35215288 10.3390/ph15020175 PMC8875116

[CIT0017] Ding L, Cui X, Jiang R, et al. (2020). Design, synthesis and characterization of a novel type of thermo-responsible phospholipid microcapsule-alginate composite hydrogel for drug delivery. Molecules 25:694. 10.3390/molecules25030694 PMC7037032 32041216

[CIT0018] Dreher MR, Liu W, Michelich CR, et al. (2006). Tumor vascular permeability, accumulation, and penetration of macromolecular drug carriers. J Natl Cancer Inst 98:335–44. 16507830 10.1093/jnci/djj070

[CIT0019] Duncan R, Connors TA, Meada H. (1996). Drug targeting in cancer therapy: the magic bullet, what next? J Drug Target 3:317–19. 8866651 10.3109/10611869608996823

[CIT0020] Fathalla D, Youssef EMK, Soliman GM. (2020). Liposomal and ethosomal gels for the topical delivery of anthralin: preparation, comparative evaluation and clinical assessment in psoriatic patients. Pharmaceutics 12:446. 10.3390/pharmaceutics12050446 PMC7285224 32403379

[CIT0021] Furst T, Dakwar GR, Zagato E, et al. (2016). Freeze-dried mucoadhesive polymeric system containing pegylated lipoplexes: towards a vaginal sustained released system for siRNA. J Control Release 236:68–78. 27329774 10.1016/j.jconrel.2016.06.028

[CIT0022] Gabizon AA, Shmeeda H, Zalipsky S. (2006). Pros and cons of the liposome platform in cancer drug targeting. J Liposome Res 16:175–83. 16952872 10.1080/08982100600848769

[CIT0023] Glavas-Dodov M, Goracinova K, Mladenovska K, et al. (2002). Release profile of lidocaine HCl from topical liposomal gel formulation. Int J Pharm 242:381–4. 12176284 10.1016/s0378-5173(02)00221-1

[CIT0024] Guan Y, Zuo T, Chang M, et al. (2015). Propranolol hydrochloride-loaded liposomal gel for transdermal delivery: characterization and in vivo evaluation. Int J Pharm 487:135–41. 25882014 10.1016/j.ijpharm.2015.04.023

[CIT0025] GuhaSarkar S, More P, Banerjee R. (2017). Urothelium-adherent, ion-triggered liposome-in-gel system as a platform for intravesical drug delivery. J Control Release 10:147–56. 10.1016/j.jconrel.2016.11.031 27913307

[CIT0026] GuhaSarkar S, Pathak K, Sudhalkar N, et al. (2016). Synergistic locoregional chemoradiotherapy using a composite liposome-in-gel system as an injectable drug depot. Int J Nanomed 11:6435–48. 10.2147/IJN.S110525 PMC5138055 27942215

[CIT0027] Haša J, Hanuš J, Štěpánek F. (2018). Magnetically controlled liposome aggregates for on-demand release of reactive payloads. ACS Appl Mater Interfaces 10:20306–14. 29791801 10.1021/acsami.8b03891

[CIT0028] Hu PZ, Zuo JF, Fu JG, et al. (2009). Preparation and antitumor immunity of long circulating nano-liposome encapsulated tumor specific antigen. Xi Bao Yu Fen Zi Mian Yi Xue Za Zhi 25:980–3. 19900361

[CIT0029] Huang Y, Hemmer E, Rosei F, et al. (2016). Multifunctional liposome nanocarriers combining upconverting nanoparticles and anticancer drugs. J Phys Chem B 120:4992–5001. 27135855 10.1021/acs.jpcb.6b02013

[CIT0030] Hurler J, Žakelj S, Mravljak J, et al. (2013). The effect of lipid composition and liposome size on the release properties of liposomes-in-hydrogel. Int J Pharm 456:49–57. 23994014 10.1016/j.ijpharm.2013.08.033

[CIT0031] Jain P, Kathuria H, Momin M. (2021). Clinical therapies and nano drug delivery systems for urinary bladder cancer. Pharmacol Ther 226:107871. 33915179 10.1016/j.pharmthera.2021.107871

[CIT0032] Jensen BE, Hosta-Rigau L, Spycher PR, et al. (2013). Lipogels: surface-adherent composite hydrogels assembled from poly(vinyl alcohol) and liposomes. Nanoscale 5:6758–66. 23685735 10.1039/c3nr01662e

[CIT0033] Kazakov S. (2016). Liposome-nanogel structures for future pharmaceutical applications: an updated review. Curr Pharm Des 22:1391–413. 26806343 10.2174/1381612822666160125114733

[CIT0034] Kiser PF, Wilson G, Needham D. (1998). A synthetic mimic of the secretory granule for drug delivery. Nature 394:459–62. 9697768 10.1038/28822

[CIT0035] Kiser PF, Wilson G, Needham D. (2000). Lipid-coated microgels for the triggered release of doxorubicin. J Control Release 68:9–22. 10884575 10.1016/s0168-3659(00)00236-4

[CIT0036] Kong Y, Dai Y, Qi D, et al. (2021). Injectable and thermosensitive liposomal hydrogels for NIR-II light-triggered photothermal-chemo therapy of pancreatic cancer. ACS Appl Bio Mater 4:7595–604. 10.1021/acsabm.1c00864 35006703

[CIT0037] Kono Y, Nakai T, Taguchi H, et al. (2017). Development of magnetic anionic liposome/atelocollagen complexes for efficient magnetic drug targeting. Drug Deliv 24:1740–9. 29141461 10.1080/10717544.2017.1402219 PMC8241088

[CIT0038] Krebs MD, Alsberg E. (2011). Localized, targeted, and sustained siRNA delivery. Chemistry 17:3054–62. 21341332 10.1002/chem.201003144 PMC7162106

[CIT0039] Lee JS, Chung D, Lee HG. (2008). Preparation and characterization of calcium pectinate gel beads entrapping catechin-loaded liposomes. Int J Biol Macromol 42:178–84. 18022686 10.1016/j.ijbiomac.2007.10.008

[CIT0040] Lee Y, Thompson DH. (2017). Stimuli-responsive liposomes for drug delivery. Wiley Interdiscip Rev Nanomed Nanobiotechnol 9:e1450. 10.1002/wnan.1450 PMC5557698 28198148

[CIT0041] Li L, Wang Q, Zhang X, et al. (2018). Dual-targeting liposomes for enhanced anticancer effect in somatostatin receptor II-positive tumor model. Nanomedicine (Lond) 13:2155–69. 30265184 10.2217/nnm-2018-0115

[CIT0042] Li R, Lin Z, Zhang Q, et al. (2020). Injectable and in situ-formable thiolated chitosan-coated liposomal hydrogels as curcumin carriers for prevention of in vivo breast cancer recurrence. ACS Appl Mater Interfaces 12:17936–48. 32208630 10.1021/acsami.9b21528

[CIT0043] Li T, Ci T, Chen L, et al. (2014). Salt-induced reentrant hydrogel of poly(-ethylene glycol)-poly(lactide-*co*-glycolide) block copolymers. Polym Chem 5:979–91.

[CIT0044] Li WZ, Zhao N, Zhou YQ, et al. (2012). Post-expansile hydrogel foam aerosol of PG-liposomes: a novel delivery system for vaginal drug delivery applications. Eur J Pharm Sci 47:162–9. 22705561 10.1016/j.ejps.2012.06.001

[CIT0045] Liu B, Sun J, Zhu J, et al. (2020). Injectable and NIR-responsive DNA-inorganic hybrid hydrogels with outstanding photothermal therapy. Adv Mater 32:e2004460. 32830376 10.1002/adma.202004460

[CIT0046] Liu W, Kong Y, Ye A, et al. (2020). Preparation, formation mechanism and in vitro dynamic digestion behavior of quercetin-loaded liposomes in hydrogels. Food Hydrocolloids 104:105743.

[CIT0047] Madan S, Nehate C, Barman TK, et al. (2019). Design, preparation, and evaluation of liposomal gel formulations for treatment of acne: in vitro and in vivo studies. Drug Dev Ind Pharm 45:395–404. 30442066 10.1080/03639045.2018.1546310

[CIT0048] Mao Y, Li X, Chen G, et al. (2016). Thermosensitive hydrogel system with paclitaxel liposomes used in localized drug delivery system for in situ treatment of tumor: better antitumor efficacy and lower toxicity. J Pharm Sci 105:194–204. 26580704 10.1002/jps.24693

[CIT0049] Matthew RA, Scott HM, Michael JM, et al. (2021). Peptide functionalized liposomes for receptor targeted cancer therapy. APL Bioeng 5:011501. 33532673 10.1063/5.0029860 PMC7837755

[CIT0050] Mehata AK, Viswanadh MK, Sonkar R, et al. (2020). Formulation and in vitro evaluation of upconversion nanoparticle-loaded liposomes for brain cancer. Ther Deliv 11:557–71. 32867624 10.4155/tde-2020-0070

[CIT0051] Meng Z, Zhang Y, She J, et al. (2021). Ultrasound-mediated remotely controlled nanovaccine delivery for tumor vaccination and individualized cancer immunotherapy. Nano Lett 21:1228–37. 33522825 10.1021/acs.nanolett.0c03646

[CIT0052] Mohammadi M, Hamishehkar H, Ghorbani M, et al. (2021). Engineering of liposome structure to enhance physicochemical properties of spirulina plantensis protein hydrolysate: stability during spray-drying. Antioxidants (Basel) 10:1953. 34943056 10.3390/antiox10121953 PMC8749985

[CIT0053] Mourtas S, Fotopoulou S, Duraj S, et al. (2007). Liposomal drugs dispersed in hydrogels: effect of liposome, drug and gel properties on drug release kinetics. Colloid Surf B Biointerfaces 55:212–21. 17223020 10.1016/j.colsurfb.2006.12.005

[CIT0054] Mourtas S, Fotopoulou S, Duraj S, et al. (2007). Liposomal drugs dispersed in hydrogels. Effect of liposome, drug and gel properties on drug release kinetics. Colloids Surf B Biointerfaces 55:212–21. 17223020 10.1016/j.colsurfb.2006.12.005

[CIT0055] Moustafa MA, El-Refaie WM, Elnaggar YSR, et al. (2018). Gel in core carbosomes as novel ophthalmic vehicles with enhanced corneal permeation and residence. Int J Pharm 546:166–75. 29778824 10.1016/j.ijpharm.2018.05.040

[CIT0056] Nasr S, Rady M, Gomaa I, et al. (2019). Ethosomes and lipid-coated chitosan nanocarriers for skin delivery of a chlorophyll derivative: a potential treatment of squamous cell carcinoma by photodynamic therapy. Int J Pharm 568:118528. 31323373 10.1016/j.ijpharm.2019.118528

[CIT0057] Noble GT, Stefanick JF, Ashley JD, et al. (2014). Ligand-targeted liposome design: challenges and fundamental considerations. Trends Biotechnol 32:32–45. 24210498 10.1016/j.tibtech.2013.09.007

[CIT0058] Onishchenko N, Tretiakova D, Vodovozova E. (2021). Spotlight on the protein corona of liposomes. Acta Biomater 134:57–78. 34364016 10.1016/j.actbio.2021.07.074

[CIT0059] Palliser D, Chowdhury D, Wang QY, et al. (2006). An siRNA-based microbicide protects mice from lethal herpes simplex virus 2 infection. Nature 439:89–94. 16306938 10.1038/nature04263

[CIT0060] Panwar P, Pandey B, Lakhera PC, et al. (2010). Preparation, characterization, and in-vitro release study of albendazole encapsulated nanosize liposomes. Int J Nanomed 5:101–8. 10.2147/ijn.s8030 PMC2841488 20309396

[CIT0061] Pérez-Herrero E, Fernández-Medarde A. (2015). Advanced targeted therapies in cancer: drug nanocarriers, the future of chemotherapy. Eur J Pharm Biopharm 93:52–79. 25813885 10.1016/j.ejpb.2015.03.018

[CIT0062] Petralito S, Spera R, Pacelli S, et al. (2014). Design and development of PEG-DMA gel-in-liposomes as a new tool for drug delivery. React Funct Polym 77:30–8.

[CIT0063] Qin C, Lv Y, Xu C, et al. (2018). Lipid-bilayer-coated nanogels allow for sustained release and enhanced internalization. Int J Pharm 551:8–13. 30196141 10.1016/j.ijpharm.2018.09.008

[CIT0064] Raemdonck K, Braeckmans K, Demeester J, et al. (2014). Merging the best of both worlds: hybrid lipid-enveloped matrix nanocomposites in drug delivery. Chem Soc Rev 43:444–72. 24100581 10.1039/c3cs60299k

[CIT0065] Rahni S, Kazakov S. (2017). Hydrogel micro-/nanosphere coated by a lipid bilayer: preparation and microscopic probing. Gels 3:7. 10.3390/gels3010007 PMC6318628 30920504

[CIT0066] Ribovski L, Zhou Q, Chen J, et al. (2020). Light-induced molecular rotation triggers on-demand release from liposomes. Chem Commun (Camb) 56:8774–7. 32618300 10.1039/d0cc02499f

[CIT0067] Ricci EJ, Lunardi LO, Nanclares DM, et al. (2005). Sustained release of lidocaine from Poloxamer 407 gels. Int J Pharm 288:235–44. 15620863 10.1016/j.ijpharm.2004.09.028

[CIT0068] Ross C, Taylor M, Fullwood N, et al. (2018). Liposome delivery systems for the treatment of Alzheimer’ s disease. Int J Nanomedicine 13:8507–22. 30587974 10.2147/IJN.S183117 PMC6296687

[CIT0069] Ruel-Gariépy E, Leclair G, Hildgen P, et al. (2002). Thermosensitive chitosan-based hydrogel containing liposomes for the delivery of hydrophilic molecules. J Control Release 82:373–83. 12175750 10.1016/s0168-3659(02)00146-3

[CIT0070] Spera R, Apollonio F, Liberti M, et al. (2015). Controllable release from high-transition temperature magnetoliposomes by low-level magnetic stimulation. Colloids Surf B Biointerfaces 131:136–40. 26042528 10.1016/j.colsurfb.2015.04.030

[CIT0071] Su J, Li J, Liang J, et al. (2021). Hydrogel preparation methods and biomaterials for wound dressing. Life (Basel) 11:1016. 34685387 10.3390/life11101016 PMC8540918

[CIT0072] Sudhakar K, Fuloria S, Subramaniyan V, et al. (2021). Ultraflexible liposome nanocargo as a dermal and transdermal drug delivery system. Nanomaterials (Basel) 11:2557. 34685005 10.3390/nano11102557 PMC8537378

[CIT0073] Sugiyama I, Ando K, Sadzuka Y. (2022). The basic study of liposome in temperature-sensitive gel at body temperature for treatment of peritoneal dissemination. Gels 8:252. 35621550 10.3390/gels8050252 PMC9141445

[CIT0074] Sung H, Ferlay J, Siegel RL, et al. (2021). Global cancer statistics 2020: GLOBOCAN estimates of incidence and mortality worldwide for 36 cancers in 185 countries. CA Cancer J Clin 71:209–49. 33538338 10.3322/caac.21660

[CIT0075] Sutoris K, Vetvicka D, Horak L, et al. (2012). Evaluation of topical photodynamic therapy of mammary carcinoma with an experimental gel containing liposomal hydroxyl-aluminium phthalocyanine. Anticancer Res 32:3769–74. 22993318

[CIT0076] T S A, Lu YJ, Chen JP. (2020). Optimization of the preparation of magnetic liposomes for the combined use of magnetic hyperthermia and photothermia in dual magneto-photothermal cancer therapy. Int J Mol Sci 21:5187. 10.3390/ijms21155187 PMC7432522 32707876

[CIT0077] Tabandeh H, Mortazavi SA. (2013). An investigation into some effective factors on encapsulation efficiency of alpha-tocopherol in MLVs and the release profile from the corresponding liposomal gel. Iran J Pharm Res 12:21–30. 24250668 PMC3813372

[CIT0079] Tanrıverdi ST, Hilmioğlu Polat S, Yeşim Metin D, et al. (2016). Terbinafine hydrochloride loaded liposome film formulation for treatment of onychomycosis: in vitro and in vivo evaluation. J Liposome Res 26:163–73. 26226352 10.3109/08982104.2015.1067892

[CIT0080] Thompson BR, Zarket BC, Lauten EH, et al. (2020). Liposomes entrapped in biopolymer hydrogels can spontaneously release into the external solution. Langmuir 36:7268–76. 32543183 10.1021/acs.langmuir.0c00596

[CIT0081] Varghese JS, Chellappa N, Fathima NN. (2014). Gelatin–carrageenan hydrogels: role of pore size distribution on drug delivery process. Colloids Surf B Biointerfaces 113:346–51. 24126319 10.1016/j.colsurfb.2013.08.049

[CIT0082] Wang X, Swing CJ, Feng T, et al. (2020). Effects of environmental pH and ionic strength on the physical stability of cinnamaldehyde-loaded liposomes. J Dispersion Sci Technol 41:1568–75.

[CIT0083] Wang Y, Wen C, Ye J, et al. (2021). Cytotoxic effect of photodynamic liposome gel combined with trastuzumab on drug resistant breast cancer cells in vitro. Nan Fang Yi Ke Da Xue Xue Bao 41:164–72. 33624588 10.12122/j.issn.1673-4254.2021.02.02 PMC7905240

[CIT0084] Wu W, Dai Y, Liu H, et al. (2018). Local release of gemcitabine via in situ UV-crosslinked lipid-strengthened hydrogel for inhibiting osteosarcoma. Drug Deliv 25:1642–51. 30799654 10.1080/10717544.2018.1497105 PMC6116704

[CIT0085] Xu XL, Huang XF, Xiao WB, et al. (2022). Preparation and pharmacological research of cetirizine hydrochloride liposome gel. J China Pharmaceuticals 12:47–52.

[CIT0086] Yan W, Leung SS, To KK. (2020). Updates on the use of liposomes for active tumor targeting in cancer therapy. Nanomedicine (Lond) 15:303–18. 31802702 10.2217/nnm-2019-0308

[CIT0087] Yang Y, Liu X, Ma W, et al. (2021). Light-activatable liposomes for repetitive on-demand drug release and immunopotentiation in hypoxic tumor therapy. Biomaterials 265:120456. 33099066 10.1016/j.biomaterials.2020.120456

[CIT0088] Yao C, Wang P, Li X, et al. (2016). Near-infrared-triggered azobenzene-liposome/upconversion nanoparticle hybrid vesicles for remotely controlled drug delivery to overcome cancer multidrug resistance. Adv Mater 28:9341–8. 27578301 10.1002/adma.201503799

[CIT0089] Yatvin MB, Weinstein JN, Dennis WH, et al. (1978). Design of liposomes for enhanced local release of drugs by hyperthermia. Science 202:1290–3. 364652 10.1126/science.364652

[CIT0090] Yazar S, Lin CH, Wei FC. (2004). One-stage reconstruction of composite bone and soft-tissue defects in traumatic lower extremities. Plast Reconstr Surg 114:1457–66. 15509933 10.1097/01.prs.0000138811.88807.65

[CIT0091] Yoon HY, Chang IH, Goo YT, et al. (2019). Intravesical delivery of rapamycin via folate-modified liposomes dispersed in thermo-reversible hydrogel. Int J Nanomedicine 14:6249–68. 31496684 10.2147/IJN.S216432 PMC6689153

[CIT0092] Youssef H, Neeshma D, Juewen L. (2013). Electrostatically directed liposome adsorption, internalization and fusion on hydrogel microparticles. Soft Matter 9:6151.

[CIT0093] Yue W, Chen L, Yu L, et al. (2019). Checkpoint blockade and nanosonosensitizer-augmented noninvasive sonodynamic therapy combination reduces tumour growth and metastases in mice. Nat Commun 10:2025. 31048681 10.1038/s41467-019-09760-3 PMC6497709

[CIT0095] Zhang J, Chen X, Xue T, et al. (2020). Liposomes encapsulating neoantigens and black phosphorus quantum dots for enhancing photothermal immunotherapy. J Biomed Nanotechnol 16:1394–405. 33419493 10.1166/jbn.2020.2977

