# Supplementary material for: Design and applications of liposome-in-gel as carriers for cancer therapy
Source: Drug Deliv. 2022 Oct 30;29(1):3245–55. doi: 10.1080/10717544.2022.2139021 (PMC9629847; doi:10.1080/10717544.2022.2139021)
Supplement: Supplemental Material [file IDRD_A_2139021_SM4751.docx]

**Table** S1. Clinical trials of LP-Gel hybrids

| Clinical Trials | Diseases | LP-Gel Hybrids | Phase |
| --- | --- | --- | --- |
| Topical Liposomal Amphotericin B Gel Treatment for Cutaneous Leishmaniasis | Leishmaniasis | Topical Amphotericin-B 0.4% liposomal gel | Phase 2 |
| Phase 2 Study of HL-009 Liposomal Gel to Treat Mild to Moderate Atopic Dermatitis | Atopic Dermatitis | HL-009 Liposomal Gel | Phase 2 |
| Liposomal Lidocaine Gel for Oral Topical Anesthesia | Anesthesia | Lidocaine | Phase 1 |
